# A Revolution Necessary and Desirable

**Published:** 1906-04

**Authors:** 


### A Revolution Necessary and Desirable.

Throughout the length and breadth of this vast land of ours, the members of the American Medical Association are gradually awakening to a realization and appreciation of "Machine" policies and politics, and, consequently, are beginning to ask pertinent questions concerning the general management of their great organization.

It is becoming more apparent with the days that pass, that there are many things requiring explanation, and there is an under-current of dissatisfaction that threatens to become an actual revolt at the very evident present tendency towards "Boss Rule" in association affairs.

Efforts to secure more explicit details in regard to the financial expenditures of the Trustees were shelved with temporary safety at Portland last July; but it remains to be seen whether or not the same opportune disposition of embarrassing questions can be made at Boston next June.

It is beyond question that the income from the *Journal* in even its present expurgated form, is exceedingly large. While it is not entirely clear whether membership in the Association goes with the *Journal*, or whether the *Journal* goes with membership in the Association, the fact remains that the gross receipts from both combinations can not be very far from *two hundred and fifty thousand dollars annually!*

But—who gets the profits?

The annual meeting certainly costs the Association little or nothing, for the expense attending it is always met by the local committee, and the locality in which it is held. The Association is doing little or nothing to financially assist medical research. There are no endowments for professorships in leading institutions; neither are there any scholarships for post-graduate or foreign study. In fact, there are no expenditures for an hundred and one different purposes that a great, scientific organization could and should properly support.

Where, then, do the profits go?

There can be but one answer—*salaries*, SALARIES, SALARIES—for the Lord alone knows who and for the Lord alone knows what; for traveling representatives whose sole aim and object is to bring "sheep into the fold" at five dollars per head; in lobbying; "in paths that are dark and ways that are hidden," in—but what's the use?

Any intelligent person who stops to consider the matter, and who has followed the Association since the death of the late lamented Hamilton, must realize that the Association, as at present conducted, is very far from first principles, to say the least.

Right here let us reiterate that we are not criticising the great American Medical Association *as a body*. We feel that it is a noble organization, with remarkable possibilities for good; that it can be a power in the betterment and progress of the American medical profession and in a closer approach to medical ideals. But the element of "graft" must be removed from its fundamental management and, in place of men who will use its great influence for sordid personal ends and glittering ambitions, must be installed men of broad character, scientific attainments, and noble purposes. Politicians are not desirable for such positions, for politicians are much like pathogenic bacteria,—they live at the expense of those they afflict.

A little while longer the members of the Association will allow the present methods of management to continue. Then the rumblings of dissatisfaction will change to action; investigations and inquiries will follow and, like the spectacle to which we have been treated in the cases of our large insurance companies, a few more damaged reputations will seek oblivion in welcome retirement.

Purged of its evils, the American Medical Association and its organ—the *J. A. M. A.*—can be made thoroughly representative of American medicine. The *Journal* may then become what thousands of medical men earnestly desire to see it—a weekly installment of the transactions of the Association, with such news, notes, information and reports as are of importance to members and, generally, the medical profession of the United States of America. In no sense will the *Journal* be a competitor of the great American medical weeklies. Instead, it will be in a distinct class by itself, an official organ complete in organization matters and medical affairs and able to further the national progress of a united profession in every particular. It will correlate, and not supersede, the independent medical publications of the country that have a legitimate field, and leave them to win the measure of success they actually deserve. The great profits from the Association may then be diverted to channels that will at once suggest themselves, in the uplifting of the medical sciences.

When, therefore, the turmoil and struggle of desirable revolution have passed away, the Association, its *Journal* and its Managing Board can truly aim—first, last and always—to be constructive, building up the sciences as exemplified by American practitioners of medicine, honestly raising the ideals and standards of practice, and freeing the noble art of medicine from the fetichism of ignorance, bigotry and tradition. Then—and not until then—will the American Medical Association come into its own.

Meanwhile, questions are in order and from time to time we shall submit a few that seem relevant and proper.—*Editorial in American Medical Journalist,* December, 1905.

